# Population genetic structure of Guiana dolphin (*Sotalia guianensis*) from the southwestern Atlantic coast of Brazil

**DOI:** 10.1371/journal.pone.0183645

**Published:** 2017-08-24

**Authors:** Juliana Ywasaki Lima, Filipe Brum Machado, Ana Paula Cazerta Farro, Lupércio de Araújo Barbosa, Leonardo Serafim da Silveira, Enrique Medina-Acosta

**Affiliations:** 1 Laboratory of Morphology and Animal Pathology, Universidade Estadual do Norte Fluminense Darcy Ribeiro, Campos dos Goytacazes, Rio de Janeiro, Brazil; 2 Laboratory of Biotechnology, Universidade Estadual do Norte Fluminense Darcy Ribeiro, Campos dos Goytacazes, Rio de Janeiro, Brazil; 3 Laboratory of Genetics and Animal Conservation, Universidade Federal do Espírito Santo, São Mateus, Espírito Santo, Brazil; 4 Institute Organization and Environmental Consciousness—ORCA, Vila Velha, Espírito Santo, Brazil; National Cheng Kung University, TAIWAN

## Abstract

*Sotalia guianensis* is a small dolphin that is vulnerable to anthropogenic impacts. Along the Brazilian Atlantic coast, this species is threatened with extinction. A prioritized action plan for conservation strategies relies on increased knowledge of the population. The scarcity of studies about genetic diversity and assessments of population structure for this animal have precluded effective action in the region. Here, we assessed, for the first time, the genetic differentiation at 14 microsatellite loci in 90 *S*. *guianensis* specimens stranded on the southeastern Atlantic coast of the State of Espírito Santo, Brazil. We estimated population parameters and structure, measured the significance of global gametic disequilibrium and the intensity of non-random multiallelic interallelic associations and constructed a provisional synteny map using *Bos taurus*, the closest terrestrial mammal with a reference genome available. All microsatellite loci were polymorphic, with at least three and a maximum of ten alleles each. Allele frequencies ranged from 0.01 to 0.97. Observed heterozygosity ranged from 0.061 to 0.701. The mean inbreeding coefficient was 0.103. Three loci were in Hardy-Weinberg disequilibrium even when missing genotypes were inferred. Although 77 of the 91 possible two-locus associations were in global gametic equilibrium, we unveiled 13 statistically significant, sign-based, non-random multiallelic interallelic associations in 10 two-locus combinations with either coupling (*D*' values ranging from 0.782 to 0.353) or repulsion (*D*' values -0.517 to -1.000) forces. Most of the interallelic associations did not involve the major alleles. Thus, for either physically or non-physically linked loci, measuring the intensity of non-random interallelic associations is important for defining the evolutionary forces at equilibrium. We uncovered a small degree of genetic differentiation (F_ST_ = 0.010; *P*-value = 0.463) with a hierarchical clustering into one segment containing members from the southern and northern coastal regions. The data thus support the scenario of little genetic structure in the population of *S*. *guianensis* in this geographic area.

## Introduction

The Guiana dolphin, *Sotalia guianensis*, is a small dolphin of the Delphinidae family [1], distributed primarily along the tropical and subtropical Atlantic coast of South and Central America [1, 2]. The north and south limit records are in La Mosquitia, Honduras, and Florianópolis, Brazil, respectively [3, 4]. There are records from Central to South America including Nicaragua, Costa Rica, Panama, Venezuela [5], Colombia [6], Guiana [7], Suriname [8], French Guiana [9] and Trinidad and Tobago [10]. Despite being distributed in the coastal region, the Guiana dolphin is commonly found in more protected areas, such as estuarine and bay regions [11]. There is no evidence of significant discontinuity in its distribution, although in many regions individuals are rarely seen; they have not been observed in some areas, but they have never been specifically sought there [12].

The Brazilian Institute of the Environment and Renewable Natural Resources stated through the Brazilian Aquatic Mammals Action Plan that knowledge of the population genetic diversity of cetaceans is a priority for the development of management and protection strategies [12]. In 2012, the International Union for Conservation of Nature (IUCN) recommended, as a conservation priority, the assessment of genetic diversity of species [13]. Notably, the IUCN classified *S*. *guianensis* as a data deficient (DD) species, reflecting the scarcity of studies on anthropogenic impacts. In 2014, the Brazilian Ministry of the Environment included *S*. *guianensis* in the list of species threatened with extinction and categorized it as a vulnerable species, following the recommendation of the Chico Mendes Institute for the Conservation of Biodiversity, ICMBio, Brazil [14].

Typing nuclear DNA polymorphic loci has been widely used in population genetics in many mammals. Multiallelic microsatellite loci are the most frequently genotyped in cetacean species [15–41]. The population-genetic studies on Guiana dolphin in the coast of Brazil have been limited to two reports. Using mitochondrial DNA haplotypes, one study [33] characterized six different state management units: Pará, Ceará, Rio Grande do Norte, Bahia, Espírito Santo, and in the southeast coast from the Rio de Janeiro to Santa Catarina states. Using microsatellites, one study [34] found low genetic differentiation between populations from the states of São Paulo and Rio de Janeiro.

The aim of the present study was to assess the degree of genetic differentiation at 14 microsatellite loci in 90 specimens of *Sotalia guianensis* stranded in the southwestern Atlantic coast of the State of Espírito Santo, Brazil, a coastal region that had not previously been sampled. We uncovered a small degree of genetic differentiation and hierarchical clustering into one segment containing memberships from the south and north coastal regions. The data thus support the scenario of little genetic structure in the population in this geographic area.

## Materials and methods

### Ethics statement

Specimen collection was carried out under authorizations from the Chico Mendes Institute for the Conservation of Biodiversity–ICMBio (URL: http://www.icmbio.gov.br/portal/) with licenses #20264/2 and #29363/4 to one of the authors (LAB) from the Institute Organization and Environmental Consciousness (ORCA) headquarters in the cities of Guarapari and Vila Velha, Espírito Santo, Brazil. The ORCA and the Universidade Federal do Espírito Santo institutional boards approved the study.

### Specimens, sample collection, and DNA extraction

Ninety specimens of *S*. *guianensis* stranded on the coast of Espírito Santo, Brazil, were collected in the period 2004–2014. The study area extended from the northern part of the state, in the municipality of Conceição da Barra (18°35′34″S 39°43′55″W), to the extreme south, in the city of Presidente Kennedy (21°05′56″S 41°02′48″W). The collection localities for each specimen are provided in S1 Table. Fragments of muscle tissue were sampled at necropsy, frozen or preserved in 70% alcohol and stored at -20°C. Samples were transferred to the Genetics and Animal Conservation Laboratory of the Universidade Federal do Espírito Santo for extraction of total genomic DNA using the salting-out method [42]. DNA was quantified using a NanoDrop 2000c UV Spectrophotometer (Thermo Scientific, Wilmington, DE, USA).

### Microsatellite genotyping

A set of 14 microsatellite loci was chosen based on population parameters previously reported in genetic studies in *Sotalia* spp. (five loci [43]), *Tursiops* spp. (six loci [20, 44]), *Inia* spp. (two loci [45]) and *Megaptera* spp. (one locus [34]). Samples were genotyped for *SRY* (chromosome Y) and *ZFX/ZFY* (chromosomes X and Y) genes to score gender, using primer sequences reported in the literature [46] and further tested in this study. Alleles were amplified by Quantitative Fluorescence Polymerase Chain Reaction (QF-PCR) assays. S2 Table lists (*i*) the microsatellite repeat units reported in the literature; (*ii*) the estimated repeat unit number found in nucleotide databases using the *In-silico PCR* [47] and the *Primer-BLAST* [48] online programs, available at the University of California, Santa Cruz (UCSC) and National Center for Biotechnology Information (NCBI) genome browsers, respectively; and (*iii*) the primer pair sequences and the QF-PCR assay conditions. DNA amplification was performed in a GeneAmp® PCR System 9700 thermocycler (Applied Biosystems, Foster City, CA, USA). Typically, a reaction mixture contained 20 ng of DNA, 0.16–2.4 μM of each primer, 2 mM MgCl_2_ and 0.5 U Taq polymerase in 12.5 μL. The amplification conditions were as follows: 95°C for 11 min; 28 cycles of 95°C for 1 min, 58–59° C for 1 min, and 72° C for 1 min; and 60° C for 60 min. Amplimers were analyzed by high-performance capillary electrophoresis in an ABI 310 Genotyper (Applied Biosystems) using the POP-4 polymer. Injection reactions typically consisted of 0.55 μL of amplimer(s), 9.0 μL of Formamide Hi-Di Formamide and 0.1 μL GeneScan ™ 500LIZ® Size Standard molecular weight ladder, all reagents from Applied Biosystems. Allele profiles were analyzed using GeneMapper ID v3.2 software (Applied Biosystems). We sequenced by the Sanger method at least one amplimer for each of the Sota-10, Sota-11, Sota-12 and Sota-13 loci, microsatellite loci that had not previously been tested in *Sotalia* spp., to determine the number of repeat units.

### Microsatellite mutability estimates

Given that there is no information about the rates of mutation for any of the microsatellite loci genotyped in the present study, we used the scoring method applied for human microsatellite loci [49]. Briefly, we measured the values for four estimates of the levels of mutability that correlate positively with mutation rate: allele span, the number of alleles per locus, expected heterozygosity (H_E_) and locus diversity (*h*_locus_) [49, 50]. The values were multiplied, and the products were ranked by their ratio with the highest score. The locus diversity was calculated using the formula $h=\frac{n}{n-1}\times \left(1-\sum_{i=1}^{k}x_{i}^{2}\right)$ where *n* is the number of samples, *k* is the number of alleles, and *x*_*i*_ is the frequency of the *i*-th allele [51].

### Chromosomal coordinate conversion and synteny map

To investigate whether the microsatellite loci are linked in syntenic blocks, we first used BLAT analysis [52] with homologous and heterologous primer sets (S2 Table) to retrieve the *Tursiops truncatus* sequences from both the bulk nucleotide and reference genome reads available from the Database Resources at NCBI [53]. The structures of the repeats were determined from the ortholog sequences using the online Tandem Repeat Finder program [54]. The loci were validated computationally using the *In-Silico PCR* tool of the online visualization interface of the UCSC Genome Browser [47]. The *In-Silico PCR* tool searches a sequence database with a pair of PCR primers, using an indexing strategy for fast performance. The tool also provides the contig or chromosomal coordinates of the amplimer. The contig data were migrated from the *T*. *truncatus* assembly (Baylor Ttru_1.4/ turTru2 [55]) to the *Bos taurus* assembly (bosTau8 UMD 3.1.1 cow assembly [56]) using the *Convert* utility, which is accessed from the menu on the UCSC Genome Browser annotation tracks page. The *Convert* utility locates the position of a feature of interest in a different release of the same genome or a genome assembly of another species and provides the percent identity and the coverage in base pairs within the converted coordinates. To facilitate access to these provisional map conversions, we customized interactive sessions at the UCSC Genome Browser. The hyperlinks to the custom tracks are available in S5 Table.

### Genetic differentiation and population structure analysis

We performed a descriptive statistical analysis for all the microsatellite loci genotyped. The number of alleles per locus (*N*a), minimum and maximum frequency, observed heterozygosity (*H*_O_), expected heterozygosity (*H*_E_), polymorphic information content (PIC), and power of discrimination (PD) were calculated using Power Stat v.12 [57]. No resampled individuals were identified by comparing genotypes using the CERVUS 3.0 *software* [58]. Statistical significances of deviations from Hardy–Weinberg equilibrium (HWE) were calculated using the exact Fisher test with 30,000 shufflings (randomizations) and adjusted with the Holm-Sidak step-down method. Genotypes were tested for global gametic (linkage) disequilibrium using the Genetic Data Analysis (GDA) 1.0 software [59]. The intensity and significance of coupling and repulsion non-random multiallelic interallelic associations were determined using the Multiallelic Interallelic Disequilibrium Analysis Software *v*.1 (MIDAS) [60] according to the methodology described in [49]. The strength of sign-based overall disequilibrium for the two-locus combinations was determined using the formulas worked in Ref. [61]. Population structure analyses were performed using Wright *F* Indexes [62] and the bootstrapping method, with 30,000 random repeats and a 95% confidence interval, assuming HWE, in the GDA software [59]. Private alleles were identified using GDA. The Bayesian clustering analysis was performed using the STRUCTURE 2.3.3 computer software package [63–65]. We applied the admixture model for correlated allele frequencies (omitting the collection locations of the specimens), setting the possible number (K) of clusters from 1 to 10, with a burn-in period of 100,000 and 500,000 Markov Chain Monte Carlo (MCMC) generations and 50 iterations. Nei’s genetic distances were calculated using the GeneAlex 6.5 Office Excel extension [66], and the distance matrix was used in the MEGA V7.0.14 program [67] to generate a dendrogram by the Unweighted Pair Group Method with Arithmetic Mean (UPGMA) hierarchical clustering method.

## Results

### Population parameters and genetic diversity of microsatellite loci

**Fig 1:**
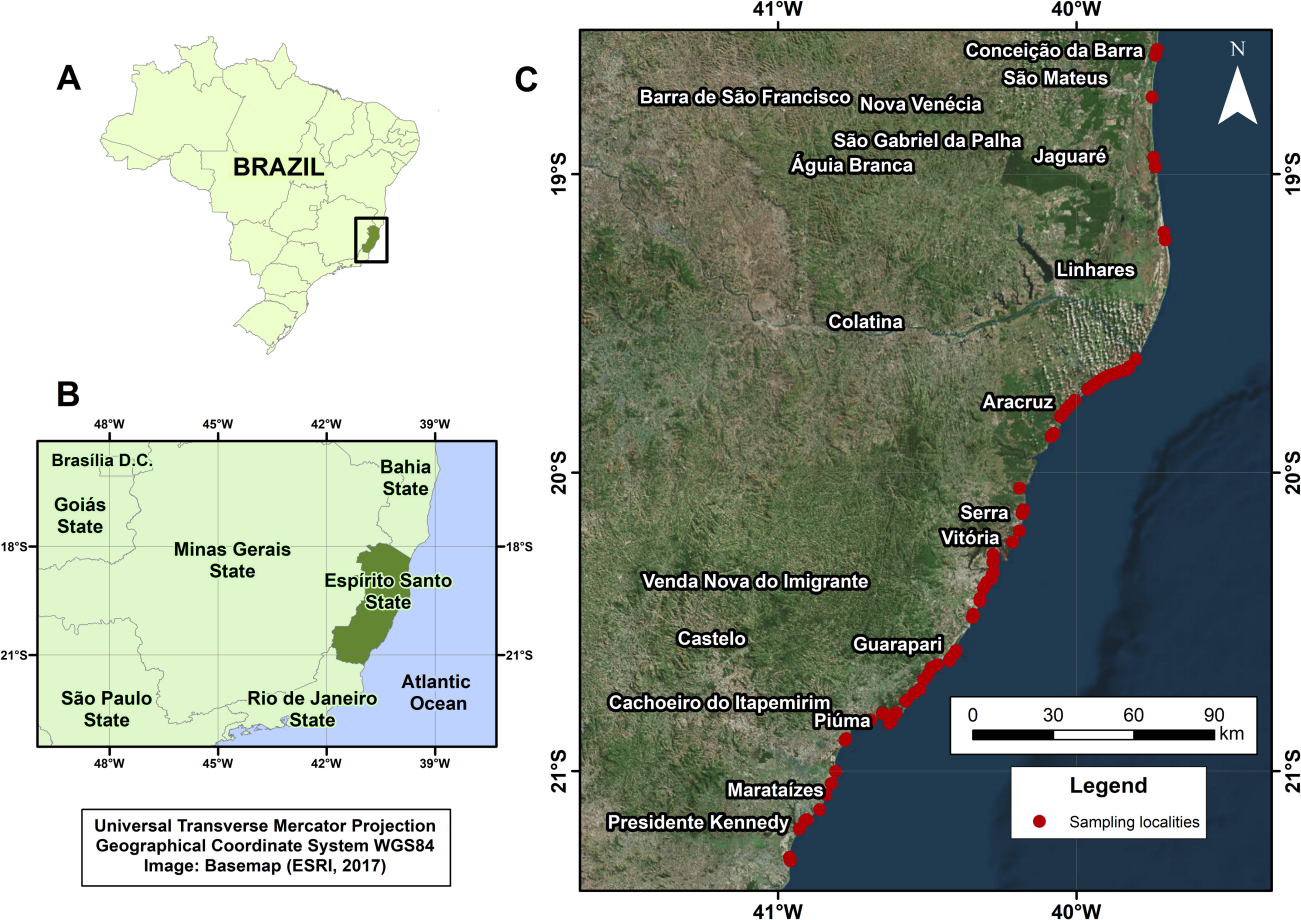
Collection localities of *Sotalia guianensis* along the coast of the State of Espírito Santo, Brazil. (A) State map of Brazil. The black rectangle indicates the State of Espírito Santo, highlighted in dark green. (B) Zoom in on image map area. (C) Range distribution of the sample localities (red dots). The dashed line represents the South and North State midline with the geographic coordinates set at 20°03'18.8"S 40°11'26.8"W.

The collection localities along the coast of the State of Espírito Santo, Brazil, are mapped in Fig 1. We typed genomic DNA samples from 90 *Sotalia guianensis* specimens with 14 microsatellite loci. The majority (69/90; 76.6%) of the biological samples were from male specimens as assessed by genotyping with DNA markers for the *ZFX/ZFY* and *SRY* genes (S2 Table). The individual genotypes are in S1 Table. Population parameters and genetic diversity estimates for the microsatellite loci are summarized in Table 1. The overall mean success rate of amplification was 70.6% (889/1,260 PCR analyses; expected rate = 90 individuals x 14 loci). All microsatellite loci were polymorphic, with at least three and a maximum of ten alleles observed per locus (mean number of alleles was 5.6). Allele frequencies ranged from 0.01 to 0.97. The most frequent alleles were Sota-11*186 (0.97) and Sota-02*208 (0.87) (S3 Table). If the specimens were to be assigned heuristically to either the southern (n = 28) or northern (n = 62) coastal regions according to the State midline, eight loci would exhibit at least one private allele, with frequencies < 0.07 (S4 Table). In this investigative scenario, 12 private alleles occurred in the specimens collected from the southern coastal region and just one from the northern area. Observed heterozygosity varied from 0.061 to 0.701, and the expected heterozygosity ranged from 0.06 to 0.81. Sota-03 exhibited the highest expected mutability level (score = 1.000), followed by Sota-12 (score = 0.908), while Sota-11 had the lowest level (score = 0.0015) (Table 2). Therefore, the least informative locus was Sota-11. The highest inbreeding coefficient *F* value was 0.266 for Sota-04, and the mean coefficient was 0.103. Three loci (Sota-01, Sota-03, and Sota-12) were in Hardy-Weinberg disequilibrium even when missing genotypes were inferred.

**Table 1:** Population parameters and genetic diversity of microsatellite loci genotyped in *Sotalia guianensis*.

	Samples	Alleles	Size range	Allele frequency							HWE (*P*-value)	
Locus	(n)	(n)	(bp)	Minor	Major	H_E_	H_O_	PIC	PD	*F*	*a*	*b*
Sota-01	70	5	131–139	0.02	0.46	0.678	0.571	0.620	0.800	0.130	0.000	0.003
Sota-02	73	5	200–228	0.01	0.88	0.237	0.260	0.210	0.400	-0.096	1.000	1.000
Sota-03	66	10	404–424	0.01	0.42	0.764	0.591	0.730	0.900	0.229	0.003	0.007
Sota-04	79	6	150–184	0.02	0.34	0.770	0.564	0.730	0.900	0.266	0.052	0.043
Sota-05	50	10	232–252	0.01	0.35	0.792	0.727	0.740	0.910	0.083	0.990	0.999
Sota-06	44	3	227–231	0.13	0.57	0.574	0.568	0.490	0.730	0.003	0.913	0.996
Sota-07	47	4	280–288	0.03	0.68	0.497	0.447	0.450	0.690	0.106	0.703	0.982
Sota-08	69	6	88–108	0.01	0.36	0.743	0.632	0.690	0.880	0.145	0.188	0.574
Sota-09	77	4	88–103	0.02	0.56	0.593	0.612	0.510	0.750	-0.054	0.998	0.995
Sota-10	71	6	210–220	0.02	0.58	0.588	0.571	0.530	0.770	0.031	0.936	0.998
Sota-11	49	3	186–206	0.01	0.97	0.060	0.061	0.060	0.120	-0.005	1.000	1.000
Sota-12	77	9	108–136	0.01	0.29	0.815	0.701	0.780	0.930	0.139	0.042	0.042
Sota-13	65	4	150–158	0.05	0.42	0.667	0.554	0.600	0.830	0.173	0.829	0.999
Sota-14	52	3	162–166	0.04	0.69	0.438	0.462	0.380	0.600	-0.091	0.988	0.999
**Mean**	**63.5**	**5.6**				**0.587**	**0.524**	**0.540**	**0.730**	**0.103**		

Number of samples *S*. *guianensis* genotyped per locus (n), number of alleles observed (n), expected (H_E_) and observed (H_O_) heterozygosity, polymorphic information content (PIC), power of discrimination (PD), inbreeding coefficient (*F*), Fisher's test *p*-value for Hardy-Weinberg equilibrium (HWE), corrected using the Holm-Sidak adjustment either disregarding (a) or inferring (b) missing genotypes.

**Table 2:** Population parameters used to estimate the levels of mutability at the 14 microsatellite loci.

Mutability parameters	Sota-01	Sota-02	Sota-03	Sota-04	Sota-05	Sota-06	Sota-07	Sota-08	Sota-09	Sota-10	Sota-11	Sota-12	Sota-13	Sota-14
Locus span (bp)	8	28	32	16	16	4	8	20	15	10	20	28	8	4
Number of alleles/locus	5	5	10	6	10	3	4	6	4	6	3	9	4	3
Expected heterozygosity	0.68	0.24	0.76	0.77	0.79	0.57	0.50	0.74	0.59	0.59	0.06	0.81	0.67	0.44
Locus diversity	0.8413	0.2941	0.8421	0.9176	0.8726	0.8511	0.6536	0.8853	0.7839	0.7004	0.0896	0.9108	0.8806	0.6510
Score value	22.81	9.76	205.97	67.79	110.60	5.86	10.39	78.96	27.89	24.71	0.32	187.03	18.80	3.42
Mutability ratio	0.1107	0.0473	1.0000	0.3291	0.5369	0.0284	0.0504	0.3833	0.1353	0.1199	0.0015	0.9080	0.0912	0.0166

We note that the Sota-10 through Sota-14 loci had not been genotyped previously in *S*. *guianensis*. To determine the number of repeat units for those microsatellite loci, we sequenced at least one allele for each locus from Sota-10 through Sota-13. The Sota-10 216 bp allele corresponds to [CA]_24_, the Sota-11 186 bp allele to [CA]_16_, the Sota-12 132 bp allele to [GT] _33_, and the Sota-13 158 bp allele to [CA]_20_.

### Chromosomal mapping of microsatellite loci by analysis of synteny

**Fig 2:**
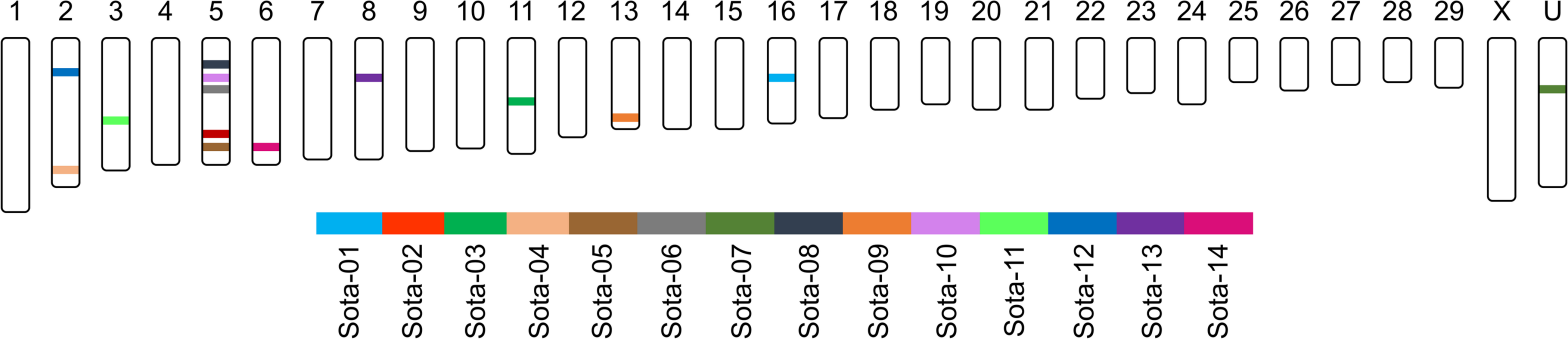
Provisional synteny map. For each *Bos taurus* chromosome, synteny segments for *Tursiops truncatus* are positionally indicated by colored bars. *Bos taurus* chromosomes are ordered by number. The U chromosome represents sequences that are unmapped to a particular chromosome.

At present, no draft of the nuclear genome sequence for *Sotalia* spp. is available for chromosomal mapping of the genetic markers used in this study. There is, however, a genome draft for the common bottlenose dolphin *Tursiops truncatus* (Baylor Ttru_1.4/ turTru2) [55]. The diploid number of chromosomes in *T*. *truncatus* is 42,XX or 42,XY [68]. No chromosomal or genetic maps are available for that species. Non-random interallelic forces among physically linked loci may influence population parameters. Therefore, to infer the physical proximity of the loci under study, we performed analysis of synteny between *Tursiops truncatus* and *Bos taurus* (bosTau8 UMD 3.1.1 cow assembly, 2009; 2n = 58,XX or 58,XY [56]) reasoning that related species are more likely to share syntenic blocks. We chose the cow assembly because it represents the reference genome available for a terrestrial mammal that is most closely related to the Delphinidae [69]. The strategy intended, first, to determine the extent of sequence homology between the *In-Silico PCR* retrieved amplimers from *Tursiops truncatus* and, second, to map by BLAT conversion the physical coordinates of the orthologous contigs in the *Bos taurus* reference genome. The orthologous contig identity ranged from 92.4% (Sota-05) to 42.2% (Sota-02) (S5 Table). Thirteen microsatellite loci were provisionally mapped in this way to the cow reference genome assembly. Five loci mapped to chromosome 5 and two others to chromosome 2. Sota-07 shares significant homology to unmapped contig sequences. The derived provisional synteny map for the microsatellite loci is shown in Fig 2.

### Global gametic disequilibrium and intensity of non-random interallelic associations

Significant global gametic disequilibrium was limited to 14 out of the 91 possible two-locus combinations (Table 3). The number of two-locus combinations varied from 13 when the missing genotypes were disregarded to 9 when they were inferred. We note that the two-locus combinations involving the microsatellite loci that are syntenic on chromosome 5 (i.e., Sota-02, -05, -06, -08 and -10) were in global gametic equilibrium. On the other hand, Sota-04 and Sota-12, which are syntenic on chromosome 2, showed global gametic disequilibrium when missing data were inferred.

**Table 3:** Two-locus combinations that exhibited significant global gametic disequilibrium.

	Global gametic disequilibrium *					
Two-locus combination	*P*-value (a)	GD	*P*-value (b)	GD	Chromosome pair	Synteny
Sota-01 / Sota-03	0.000	+	0.000	+	16/11	No
Sota-01 / Sota-04	0.049	+	0.068		16/2	No
Sota-01 / Sota-08	0.011	+	0.092		16/5	No
Sota-01 / Sota-12	0.014	+	0.041	+	16/2	No
Sota-01 / Sota-13	0.003	+	0.904		16/8	No
Sota-03 / Sota-04	0.000	+	0.000	+	11/2	No
Sota-03 / Sota-05	0.044	+	0.098		11/5	No
Sota-03 / Sota-08	0.045	+	0.046	+	11/5	No
Sota-03 / Sota-12	0.000	+	0.006	+	11/2	No
Sota-03 / Sota-13	0.000	+	0.410		11/8	No
Sota-04 / Sota-05	0.024	+	0.011	+	2/5	No
Sota-04 / Sota-08	0.000	+	0.009	+	2/5	No
Sota-04 / Sota-12	0.051		0.000	+	2/2	Yes
Sota-08 / Sota-12	0.006	+	0.017	+	5/2	No

*Significance of observed gametic disequilibrium (GD), either disregarding (a) or inferring (b) missing genotypes, estimated by Fisher exact test of independence for 30,000 runs

*P-*values > 0.000 were corrected using the Holm-Sidak adjustment.

Recombination events represent an important evolutionary process determining gametic equilibrium. Thus, we measured interallelic *D*´ coefficients between all possible two-locus combinations to uncover coupling (*D*'(+)) or repulsion (*D*'(-)) non-random interallelic forces at disequilibrium. Twelve possible two-locus combinations exhibited at least one significant interallelic association (Table 4). Thus, ten of those combinations were at apparent global equilibrium. The intensity and significance of the sign-based gametic disequilibrium and the allele pairs involved are shown in Table 4. In total, 15 statistically significant, non-random multiallelic interallelic associations were observed, 12 with coupling (*D*' values ranged 0.782 to 0.353) and 3 with repulsion (*D*' values -0.517 to -1.000) forces. Except for one allele pair in the Sota-05/Sota-13 two-locus combination, the interallelic associations did not involve the major alleles from both loci. The only syntenic two-locus non-random interallelic association observed was between Sota-02*208 bp and Sota-05*232 bp on chromosome 5, and the allele pair included the most frequent Sota-02 allele.

**Table 4:** Intensity and significance of sign-based gametic disequilibrium between two-locus combinations.

	Significance of global disequilibrium *				Intensity of sign-based disequilibrium **						
**Two-locus combination**	***P*-value** (**a**)	***P*-value** (**b**)	**Allele pair**	**Samples**	***D*'**(**+**)	**Chi-square**	**r**^**2**^	**Major allele**		**Chr. Pair**	**Synteny**
Sota-01 / Sota-03	0.000	0.000	135/436	52	0.726	7.215	0.344	No	No	16/11	No
Sota-03 / Sota-07	0.496	0.710	412/284	42	0.619	4.074	0.140	Yes	No	11/U	No
Sota-03 / Sota-12	0.000	0.006	426/134	59	0.474	5.228	0.141	No	No	11/2	No
Sota-05 / Sota-12	0.258	0.393	248/134	58	0.782	5.478	0.170	No	No	5/2	No
Sota-05 / Sota-13	0.895	1.000	232/156	54	0.560	4.819	0.131	No	No	5/8	No
Sota-05 / Sota-13	0.895	1.000	238/158	54	0.560	6.487	0.150	Yes	Yes	5/8	No
Sota-07 / Sota-12	0.966	0.985	284/134	43	0.400	4.149	0.149	No	No	U/2	No
Sota-08 / Sota-09	0.725	1.000	88/97	65	0.353	4.169	0.085	No	No	5/13	No
Sota-08 / Sota-09	0.725	1.000	96/94	65	0.353	4.762	0.114	No	No	5/13	No
Sota-08 / Sota-14	0.914	1.000	88/166	48	0.379	4.156	0.122	No	No	5/6	No
Sota-09 / Sota-10	1.000	1.000	94/218	69	0.752	5.062	0.151	No	No	13/5	No
Sota-10 / Sota-12	0.854	0.613	218/128	62	0.552	4.331	0.186	No	No	5/2	No
**Two-locus combination**	***P*-value** (**a**)	***P*-value** (**b**)	**Allele pair**	**Samples**	***D*'**(**-**)	**Chi-square**	**r**^**2**^	**Major allele**		**Chr. pair**	**Synteny**
Sota-02 / Sota-05	0.989	1.000	208/232	63	-0.517	8.526	0.197	Yes	No	5/5	Yes
Sota-03 / Sota-10	0.668	0.841	428/214	59	-1.000	4.069	0.110	No	Yes	11/5	No
Sota-08 / Sota-14	0.914	1.000	88/164	48	-0.561	8.673	0.229	No	Yes	5/6	No

* Significance of observed gametic disequilibrium, either disregarding (a) or inferring (b) missing genotypes, estimated by Fisher exact test of independence for 30,000 runs; *P*-values > 0.000000 were corrected using the Holm-Sidak adjustment.

** Sign-based intensity of significant gametic disequilibrium determined by *D*'(+) and *D*'(-) coefficients. For comparison, the r^2^ values are provided. Shown are the two-locus combinations and the allele pairs that exhibited significant associations (*P* <0.05), estimated by Yates´ chi-square test.

### Population genetic structure analysis

**Fig 3:**
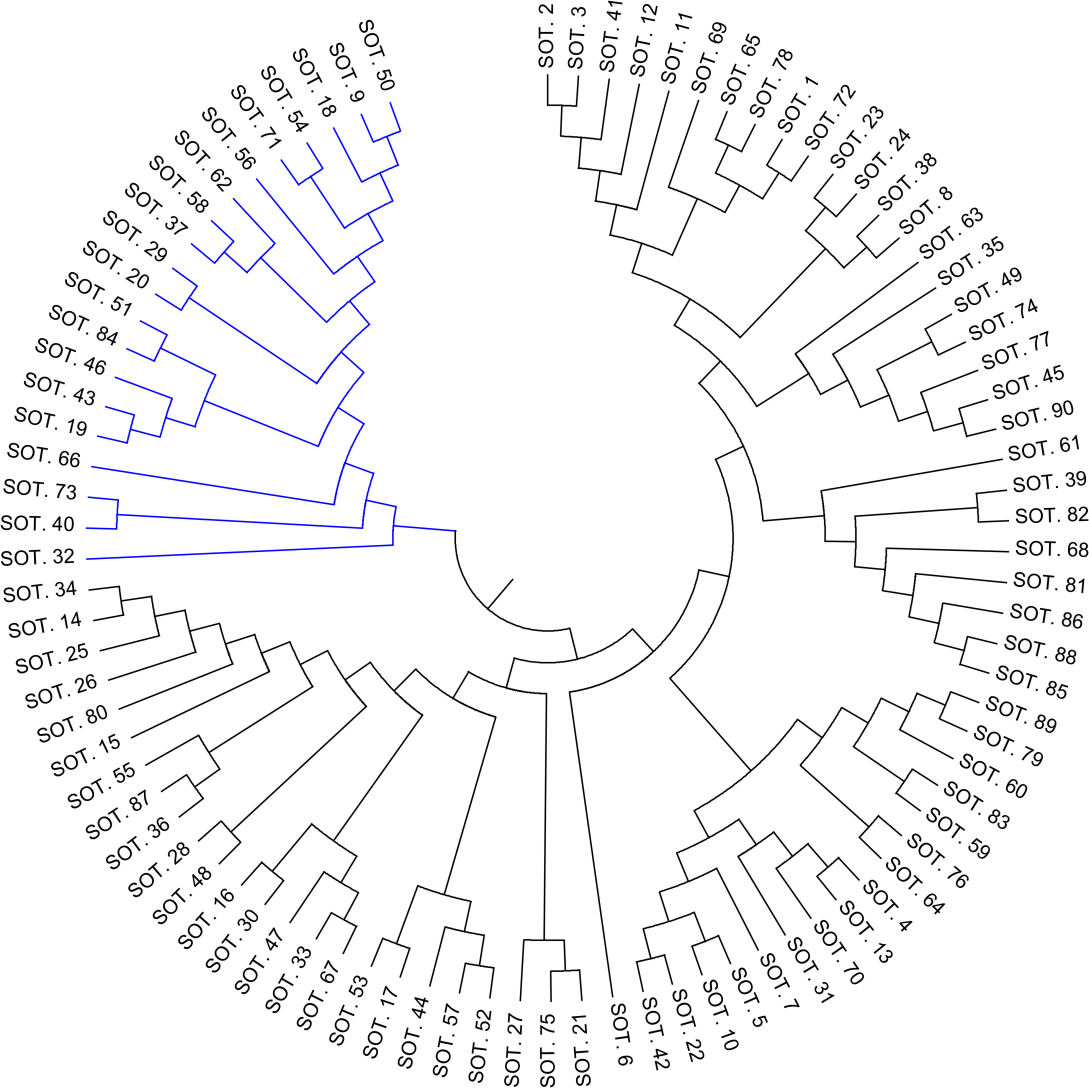
Similarity dendrogram for *Sotalia guianensis* genotypes. The similarity is based on Nei’s genetic distances at 14 microsatellite loci. The dendrogram was drawn in MEGA using the UPGMA hierarchical clustering method. The analysis indicates that the individuals were partitioned into two clusters (represented by the branches in black and blue colors), with specimens from both southern and northern coastal regions being designated to either cluster.

To evaluate the occurrence of possible patterns in the genetic composition of the 90 stranded Guiana dolphin specimens, we analyzed the genotypes in three ways with a heuristic model based on localization to designate the specimens to either the southern or northern coastal regions (Fig 1). First, we performed fixation index F-statistics to measure the degree of genetic differentiation (F_ST_ = 0.010; *P*-value = 0.463; 95%CI: -0.000–0.026, for 30,000 random replicates). Second, we employed Bayesian clustering analysis to reveal that all the genotypes clustered in one segment with no significant separation between southern and northern designations. One segment was observed by setting the possible number (K) of clusters from 1 to 10 (Posterior probabilities ranged from 1 to 0.1). Lastly, we estimated the Nei’s genetic distances at the 14 microsatellite loci, grouped the individual genotypes by similarity using hierarchical clustering, and displayed the similarity in a dendrogram (Fig 3). The analysis showed that the individuals partitioned into two hierarchical clusters. Nevertheless, both clusters comprised specimens with memberships in the southern and the northern coastal regions. There was no apparent biological aspect in the dataset that represented this hierarchical partition.

## Discussion

We show that a sampling of 90 Guiana dolphins stranded in the Atlantic coastal area of the State of Espírito Santo, Brazil, composes a population with little genetic structure. The evidence is three-fold: a low degree of genetic differentiation, low inbreeding coefficients, and clustering into one segment containing members from the southern and northern coastal regions. Our study is the first to assess the genetic diversity of *Sotalia guianensis* at microsatellite loci in this coastal area. A previous survey with 58 *S*. *guianensis* samples from the coastal areas of the States of São Paulo and Rio de Janeiro, Brazil [34], also showed low genetic differentiation coefficient (F_ST_ = 0.04) at ten microsatellites, five of which were also genotyped in our study.

Decreased locus diversity is often seen when using heterologous primer sequences (i.e., designed for one species and used in another) [70]. Here, we used nine heterologous primer sets, and only one (Sota-11) yielded low genetic diversity. Altogether, the population parameters at nine loci were consistent with the data reported in three other studies of *S*. *guianensis* that used the same primer sets [34, 43, 45]. We note that in our biological samples, the Sota-11 locus exhibited only three alleles with an allele span of 186–206 bp (equivalent to 10 [CA] repeat units). In contrast, in *T*. *truncatus*, the same locus exhibited eight alleles [20]. Our data indicate that the Sota-11 locus has the lowest estimated rate of mutability in *S*. *guianensis*.

Genetic studies in other dolphin genera (*Tursiops truncatus*, *Tursiops aduncus*, *Cephalorhynchus eutropia*, and *Stenella frontalis*) have reported F_ST_ values ranging from 0.034 to 0.20 with varying sample sizes [31, 39–41, 71] and coverages through short and long geographic distances [37, 72]. However, those values cannot be compared because they refer to species with diverse ecologies, social structures, and evolutionary histories.

The clusters created by STRUCTURE can be affected by variability in sample size [73]. We performed an average of 588 analyses (mean number of subjects scored = 42 x 14 loci) for the southern coastal population subset and 301 analyses (average number of subjects scored = 21.5 x 14 loci) for the northern population subsets. We believe, for the following reasons, that the apparent lack of structure in our population study cannot be ascribed to the small number of either individuals or loci scored. First, for microsatellite-based population genetic studies, the typing of 25 to 30 individuals per population is enough to estimate allele frequencies accurately [74]. Second, the occurrence of private alleles increases as a function of the genetic differentiation between populations [75]. When we consider the heuristic designation of the specimens to either southern or northern possible population subsets, just one private allele was detected in the northern region population subset, compared with 12 possible private alleles in the southern subgroup. The observed highly skewed distribution does not support a potential history of fragmentation and isolation.

Other factors, however, may influence the structure of a cetacean population: the distribution of prey [76, 77], social behavior [78], use of preferential habitats [79], and habitat discontinuities due to environmental characteristics [39, 80]. Unfortunately, no reports on such variables are available for the coastal region covered in our study, which impaired a fully comprehensive analysis. We note a significant (chi-squared test, *P value* = 4.20039E-07) 3-fold excess of male specimens in our samples. This imbalance may be due to anthropogenic actions, such as fishing activities. The majority of dolphins in fishing-net accidents are young and male [81], which increases the number of male animals found on beaches.

A second important aspect of our study addresses the prospective application of the syntenic map of the microsatellite loci for kinship analyses. It is evident in other biological systems [49, 82] that measuring global gametic disequilibrium alone is insufficient to define the evolutionary forces at equilibrium for either physically or non-physically linked loci. We showed that eleven of the 91 possible two-locus combinations that were in apparent global equilibrium exhibited at least one significant, sign-based non-random multiallelic interallelic association. For the five loci that are syntenic on chromosome 5, only one significant non-random interallelic association was detected, eventually compromising their combined use for estimating the power of discrimination [83]. In contrast, the two syntenic loci on chromosome 2 did not exhibit significant interallelic associations, supporting the view that these two syntenic loci may segregate independently. We therefore recommend measuring the intensity and significance of coupling and repulsion non-random multiallelic interallelic associations for future parentage-based group composition and dispersal pattern studies of cetaceans.

## Supporting information

S1 Table Geographic localities, individual genotypes and gender designations of *Sotalia guianensis* specimens. (XLSX)

S2 Table Characteristics of microsatellite loci genotyped in *Sotalia guianensis* and PCR assay conditions. (XLSX)

S3 Table Distribution of microsatellite allele frequencies observed in *Sotalia guianensis*. (XLSX)

S4 Table Private microsatellite alleles observed in *Sotalia guianensis*. (XLSX)

S5 Table Coordinate conversion and chromosomal mapping of microsatellite orthologs in *Bos taurus* reference assembly genome. (XLSX)
